# HIV-1 gp120 Protein Activates Cyclin-Dependent Kinase 1, a Possible Link to Central Nervous System Cell Death

**DOI:** 10.3390/v14122793

**Published:** 2022-12-15

**Authors:** Adonira Saro, Zhaolin Gao, Piniel Alphayo Kambey, Min Li, Jufang Huang

**Affiliations:** 1 Department of Anatomy and Neurobiology, School of Basic Medical Science, Central South University, Changsha 410013, China; 2 Xuzhou Key Laboratory of Neurobiology, Department of Neurobiology and Anatomy, Xuzhou Medical University, Xuzhou 221004, China; 3 School of Life Sciences, Central South University, Changsha 410013, China

**Keywords:** HIV-1, gp120, HAND, cell death, apoptosis

## Abstract

Human immunodeficiency virus-1 (HIV-1)-associated neurodegenerative disorder (HAND) is frequently reported in HIV-infected individuals. The gp120 envelope viral protein has been implicated in the pathogenesis of HAND in HIV-1-infected patients; however, its pathogenic mechanism remains unclear. In this study, we first overexpressed gp120 proteins in pc12 cells and used PI staining, a CCK8 assay, a TUNEL assay, and caspase-9/caspase-3-induced apoptosis to ascertain the mediated cell death. Subsequently, the gp120-overexpressed cells were subjected to RNA transcriptomics and mass spectrometry. The obtained results were integrated and validated using a quantitative polymerase chain reaction (qPCR) and the postmortem brain samples with HIV-associated dementia were analyzed against the normal control (using the GSE35864 data set on gene ontology omnibus repository). Upon the integration of the RNA transcriptomic and proteomic results, 78 upregulated genes were revealed. *Fut8*, *Unc13c*, *Cdk1*, *Loc100359539*, and *Hspa2* were the top five upregulated genes. Upon the analysis of the GSE35864 data set, the results indicate that *Cdk1* was upregulated in HIV-associated dementia in comparison to the normal control. Moreover, the protein expression of *Cdk1* was significantly higher in the gp120 transfected group compared to the normal control and decreased significantly upon inhibition using Roscovitine (a known *Cdk1* inhibitor). Taken together, our results provide a possible molecular signature of the neurological impairment secondary to HIV glycoprotein 120.

## 1. Introduction 

HIV-1 is a lentivirus of the Retroviridae family [1] and the causative agent of acquired immune deficiency syndrome (AIDS) [2]. HIV-1 infection induces a neurocognitive disorder, known as the AIDS dementia complex [3], as well as HIV-1-associated neurocognitive disorder (HAND). These two disorders occur in more than 50% of all HIV-1-infected individuals [4,5,6]. HIV-1 invades the brain cells soon after systemic infection via three basic methods: transport by infected cells (“Trojan horse” hypothesis); transfer of cell-free virus into the brain; and viral release into the brain by infected endothelial cells [7]. HIV-1 infected astrocytes may spread the HIV infection in the brain and induce damage via a gap-junction-mediated mechanism [8]. Newly infected cells continue to release viral proteins and lead to inflammation [9]. Despite the presence of surface receptors on neurons, HIV-1 does not infect them [10]. There is limited evidence that HIV-1 can directly infect neurons [11]; however, evidence from several studies support HIV-1′s indirect effects on neurons [11,12]. Several reports show the presence of HIV-1 nucleic acids in neurons extracted from a subset of AIDS patients [11,13]. Cytokines and chemokines derived from host cells are released by infected non-neuronal cells, and likely affect a diverse range of neuronal populations in the Central Nervous System (CNS) [14]. HIV-1 causes neuronal damage, cell death, and CNS dysfunction via soluble viral proteins rather than by a productive viral infection [11]. The resulting neuronal damage occurs through apoptosis and the loss of dendritic structures, and finally hastens the onset of HAND [12]. Structural proteins (Pol, Gag, and Envelope for example gp120), essential regulatory proteins (Tat and Rev), and accessory proteins (Vif, Vpr, Vpu, and Nef) constitute the HIV-1 proteins [15]. These proteins have been linked to HIV-1-associated dementia and encephalitis [16]. To cause apoptosis, HIV-1 viral proteins, including Tat, Vpr, Nef and gp120, disrupt multiple CNS functions, including chemokine synthesis, glutamate transport, and cellular pathways [17,18,19].

Glycoprotein 120 (gp120) is a component of the HIV-1 outer envelope and is essential for viral infection as it enables the entry of HIV-1 into host cells [18]. It is a major HIV-1 protein that causes neuronal damage and death in individuals with HAND [17,18]. The mechanism underlying the role of gp120 in the pathogenesis of HAND has been previously reported, yet seems to be perplexing. Ankit et al. revealed that gp120 viral proteins cause programmed cell death via endoplasmic reticulum stress [19,20]. Furthermore, gp120 causes neuronal apoptosis via RNA-activated protein kinase signaling, and stimulates IL-6 and IL-8 expression via a nuclear factor-kappa B-dependent pathway [21]. Here, we have explored the possible molecular signature whereby gp120 induces programmed neuronal cell death and validated this using the available data set of postmortem human brain samples. Our results reveal genes that possibly mediate the neuronal death in individuals with HIV presenting with neurocognitive disorders. Upon validation using postmortem human brain samples, *CDK1* tended to be upregulated in individuals with HIV-associated dementia in comparison to the normal control. On *CDK1* inhibition, the mediated markers of apoptosis decreased significantly compared to non-inhibited group.

## 2. Materials and Methods

### 2.1. Cell Cultivation

The PC12 cells were purchased from the American Type Culture Collection (ATCC), cultured in Dulbecco’s modified Eagle medium (DMEM) and supplemented with 1% penicillin–streptomycin and 10% fetal bovine serum ((FBS); Thermo Fisher Scientific, Carlsbad, CA, USA), and maintained at 37 °C in a humidified atmosphere of 5% CO_2_.

### 2.2. Cell Transfection

Two plasmids, HIV-1gp120 and an empty vector that was used as a negative control, were produced by the Zorin Biotechnology Co., Ltd., Shanghai, China. The HIV-1gp120 plasmid was transfected into PC12 cells, in strict accordance with the polyethylenimine (PEI) guidelines (Taipei, Taiwan, China). The cells (triplicate or quadruplicate wells) were transfected with HIV-1gp120 for 0–48 h (1, 2, or 3 µg/µL) in six-well plates. The same concentrations and incubation conditions were applied for the control group. All of the disposable plastic ware was procured from Thermo Fisher Scientific, Carlsbad, CA, USA.

### 2.3. CCK8 Assay

The Cell Counting Kit 8 (CCK-8 kit; Beyotime Biotechnology Co., Ltd., Shanghai, China) was used to determine cell viability. The PC12 cells were seeded at 5 × 10^3^ cells/well onto a 96-well plate and transfected with gp120 as per the PEI protocol. At 48 h post transfection, a mixture of CCK-8 reagent and DMEM (1:10) *v*/*v* was added to the cells and incubated for 30 min. Optical density (OD) was measured at 450 nm and the cell survival rate was calculated.

### 2.4. TUNEL Assay

The cultured neurons, treated with gp120, were washed in phosphate-buffered saline (PBS) and then fixed in 4% paraformaldehyde on a cover slip; 1 µL TdT enzyme, 10 µL buffer, and 5 µL Bright red dye from a TUNEL kit (Vazyme biotech company limited Nanjing China) were added to the cover slip and incubated for 1 h at 37 °C. The reaction was terminated by adding PBS to the plate and was then washed with PBS through mechanical shacking, three times, for 30 min at room temperature. To determine their nuclear morphology, the cells were counterstained with 4′,6-diamidino-2-phenylindole (DAPI; Bio-Rad, Hercules, CA, USA) and examined under a fluorescence microscope.

### 2.5. Western Blotting Analysis

The protein content was determined by BCA-assay (Thermo Fisher Scientific, Waltham, MA, USA); 20 μg total protein were resolved on 4–10% Bis-Tris gels. Standard Western blotting techniques were used with antibodies, including Flag (1:1000), Bax (1:2000), bcl2 (1:1000), and caspase 3 (1:500) (all from Proteintech, Wuhan, People’s Republic of China). Semi-quantitative analysis was performed with Image J.

### 2.6. RNA Preparation and Transcriptomics Libraries

According to the manufacturer’s instructions, the total RNA was extracted with TRIzol. A nano Drop 2000 spectrophotometer was used to measure the RNA purity and quantity (Thermo Fisher Scientific, USA). The Agilent 2100 Bio analyzer was used to test the RNA integrity (Agilent Technologies, Santa Clara, CA, USA). The libraries were created using Illumina’s TruSeq Stranded mRNA LT Sample Prep Kit (San Diego, CA, USA). The sequencing and analysis were performed by Shanghai Oe Biotech Co., Ltd., China. The detailed protocol for RNA extraction and all transcriptomics that were followed by the company are provided in Supplementary File S1.

### 2.7. Sample Preparation and Mass Spectrometry

Following the transfection of gp120 into PC12 cells for 48 h, the medium was pipetted off from the adherent cells. The surface of the culture dish was lightly scraped with sterile PBS, and the cells were then gently rinsed for 1 min before the entire solution was removed. Using a clean scraper, the cells were removed from one side of the dish and quickly transferred to the centrifuge tube. The remaining cells were cleaned with PBS before being moved to the centrifuge tube, and the precipitate was collected after centrifugation. The cellular precipitate was placed in −80 °C storage after being submerged for 5 min in liquid nitrogen. On the following day, the cells were transferred on dry ice for mass spectrometry at OE Biotech Co., Ltd. in Shanghai, People’s Republic of China. The detailed protocol for protein extraction and proteomics is provided in Supplementary File S2.

### 2.8. Selection of the Candidate Reference Genes

Venn software (https://bioinfogp.cnb.csic.es/tools/venny/index.html; accessed on 1 August 2022) was used to identify significantly upregulated overlapping genes (candidate genes) between the transcriptomics and proteomics. Panther software (http://pantherdb.org/; accessed on 1 August 2022) was used to categorize these genes based on their pathways. Five upregulated genes with FC ≥ 1.2 (*Fut8*, *Unc13c*, *Cdk1*, *LOC100359539*, and *Hspa2)* were identified as candidate genes that are associated with neurodegeneration. After selection, a quantitative polymerase chain reaction (qPCR) was performed for gene validation.

### 2.9. Isolation of RNA and cDNA Synthesis

TRIzol Reagent was used to isolate and purify the RNA from the cultured PC12 cells that express HIV-1gp120. Accordingly, using the Hifair^®^ III 1st Strand cDNA Synthesis Kit (gDNA digester plus) from Yeasted Biotechnology in Shanghai, People’s Republic of China, 3 μg total RNA were reverse-transcribed to produce cDNA. The Novo Start^®^ SYBR qPCR Super Mix plus (Novo protein, Shanghai, People’s Republic of China) was used for quantitative real-time PCR, according to the manufacturer’s protocol. With GAPDH as an internal reference, the 2^−△△Ct^ method technique was used to compute the relative gene mRNA levels for each target gene. The list of all genes and their respective primer’s sequence are provided in Table 1.

### 2.10. Bioinformatics Analysis of HIV-Associated Dementia against the Normal Control in GSE35864 Data Set

In order to achieve this, we searched for HIV patients with neurological impairment from the gene expression omnibus (GEO) database using the search term ‘Dementia in patients with HIV’ [22]. Thirteen results were obtained. After filtering these results by selecting only humans, eight results remained. After a thorough check, only one study fit the criteria (GSE35864). The GSE35864 data set was deposited by Jessica Winkler, and it contains 72 samples from different brain regions. To analyze these data, we used GEOR (https://www.ncbi.nlm.nih.gov/geo/geo2r/; accessed on 1 October 2022), an online tool that leverages the Limma package in R to compute the differentially expressed genes. The genes that were differentially expressed between HIV-associated dementia and the normal control were identified. Positive (+) log2FC genes were regarded as upregulated, while negative (−) log2FC genes were considered downregulated. The results were visualized in a Uniform Manifold Approximation and Projection plot (UMAP), Volcano plot, and mean-difference plot.

### 2.11. Pharmacological Inhibition of CDKI-Gene

After 48 h, the PC12 cells were transfected with gp120 and plated in a six-well plate. Following two washes with sterile PBS, the cells were replaced with fresh media. The cells were treated with 10 µM and 50 µM of Roscovitine [23] (Tocris Bioscience, Bristol, united Kingdom). Cells expressing gp120 without inhibitor were regarded as the control. After 12 h of treatment with CDK1 inhibitor, the cells were collected and lysed for Western blotting.

### 2.12. Statistical Analysis

Each experiment was carried out at least three times. All findings were presented as the mean ± SD. The unpaired, two-tailed Student’s *t*-test was used to compare two independent groups, and Ordinary one-way ANOVA was performed to compare multiple groups. Graph Pad Prism 7.04 was used for all statistical analyses. *p* ˂ 0.05 was considered statistically significant.

## 3. Results

### 3.1. Transfection of HIV-1 gp120 into PC12 Cells

**Figure 1:**
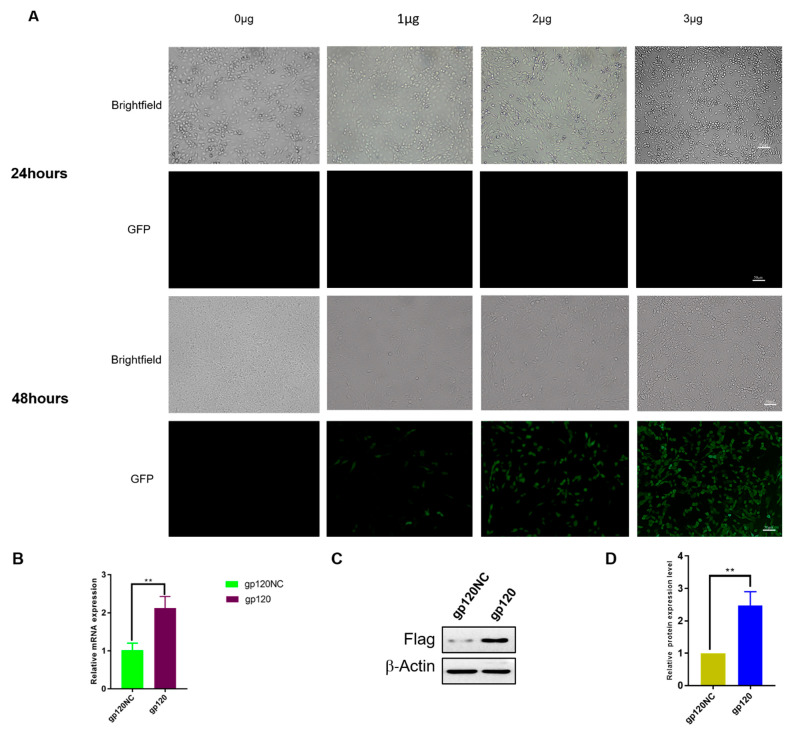
(**A**) Fluorescence microscope images of the PC12 cells displaying Green Fluorescence Plasmid (GFP)-gp120 protein. After 48 h, the PC12 cells were exposed to various doses (1 µg, 2 µg, 3 µg) of the gp120 protein coupled with the matching empty vector as a gp120 negative control (gp120NC). GFP was expressed more strongly in PC12 cells transfected with the 3 µg concentration of gp120 than with the other concentrations. (**B**) After immunofluorescence, the expression of 3 µg gp120, which was transfected in the PC12 cell line, the same batch of samples were harvested extracted for mRNA and revealed significant expression by qPCR in comparison to the gp120 negative control (gp120NC). (**C**) Again, samples with similar conditions were lysed for Western blotting in order to measure the expressed protein levels between the control group and the treatment group. (**D**) the treatment group showed significantly increased gp120 protein expression than the control group, gp120NC = glycoprotein 120 Negative control, with *p* = 0.0038. Β-actin was used as loading control. ** *p*  <  0.01, *t*-test, *n*  =  3.

To establish the model for the subsequent experiments, gp120 was transfected into the PC12 cell line, which is a prominent cell line used to explore CNS diseases [24]. We transfected PC12 cells with different concentrations of plasmid overexpressing gp120 (1, 2, and 3 μg) and detected the result at 24 and 48 h post transfection, with successful transfection indicated by GFP-positive PC12 cells. The highest transfection efficiency can be obtained with 3 μg plasmid transfection for 48 h (Figure 1A). We isolated and extracted the RNA from the cells and performed qPCR quantification. There was significant expression of gp120 mRNA in the PC12 cell-overexpressed gp120 group (Figure 1B). The Flag expression, which is integrated with gp120-overexpressing plasmid, was measured using Western blotting in the gp120-overexpressing- and empty plasmid-transfected PC12 cell group. The Western blot results revealed significant gp120 expression in the PC12 cells (Figure 1C,D).

### 3.2. HIV-1 gp120 Induced Apoptosis of PC12 Cells

**Figure 2:**
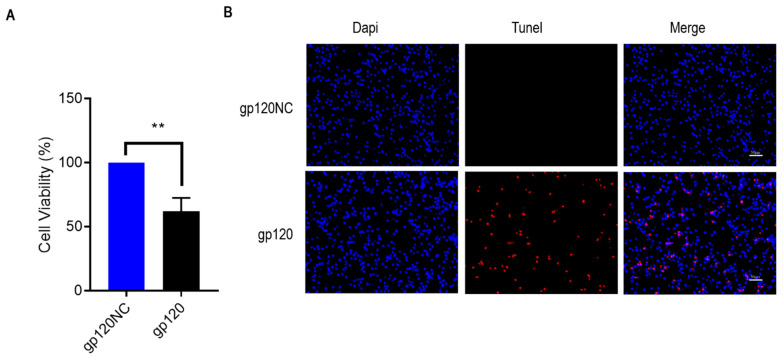

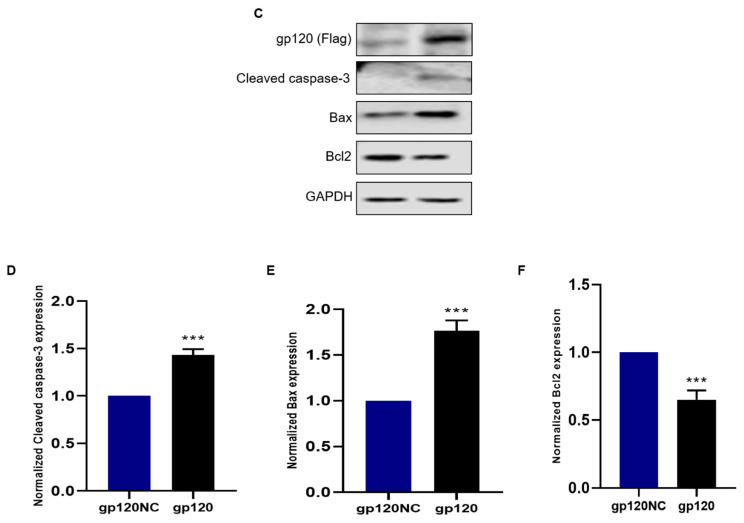
(**A**) After 48 h of PC12 transfection with gp120 plasmid and its co-corresponding empty vector, CCK8 was carried out; the statistical analysis between treatment and control group was ** *p*  <  0.01, *t*-test. (**B**) TUNEL assay and DAPI stains were used to detect apoptotic cells, as shown in the immunofluorescence images. DAPI labeling of nucleic acids was used to observe the intact cell nuclei, while TUNEL staining of fragmented DNA was used to mark apoptotic cells. (**C**) Expression of Cleaved caspase-3, Bax, and Bcl2 levels mediated by gp120 were measured and the protein levels were normalized by GAPDH and quantified by Image J. (**D**–**F**) The expression level was statistically assessed using *t*-test, *** *p* < 0.001, *n*  =  3.

The PC12 cells were transfected with 3 µg gp120-overexpressing plasmid and the CCK8 assay was performed 48 h later. The results indicate that, compared with the negative control group, the cell proliferation in the experimental group was profoundly reduced (Figure 2A). Subsequently, to detect whether gp120 overexpression in PC12 cells induces apoptosis, we performed a TUNEL assay in the two study groups. The results showed that 48 h after treatment, the 3 µg gp120 plasmid treatment induced PC12 cell apoptosis (Figure 2D). Subsequently, Western blotting was performed to further detect the expression level of apoptosis-related proteins. Compared with the control group, the levels of pro-apoptotic proteins, including cleaved caspase-3 (Figure 2D) and Bax (Figure 2E), increased, and the levels of the anti-apoptosis protein Bcl2(Figure 2F) decreased in the experimental group. This indicate that HIV-1 gp120 induces apoptosis in PC12 cells.

### 3.3. RNA Transcriptomics Reveals Genes Linked to Neurodegenerative Diseases

**Figure 3:**
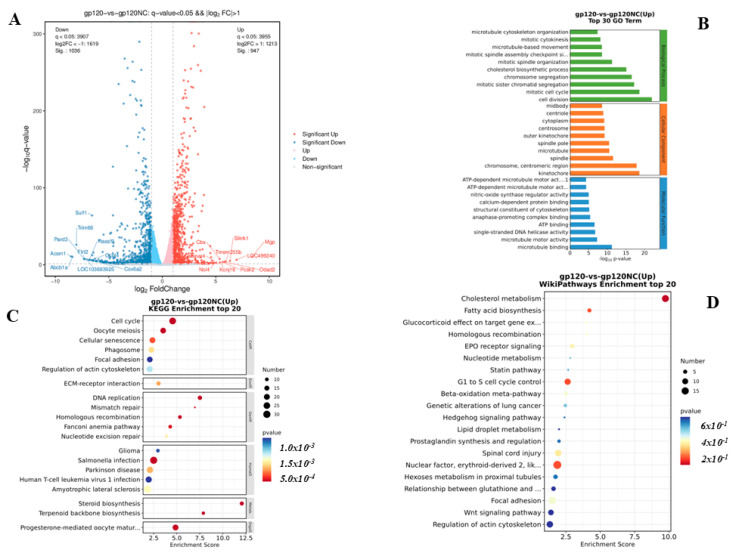
Volcano plot of DEG, GO functional analysis of DEG, KEGG pathway and wiki pathways enrichment analysis of transcriptomics PC12 cells samples overexpressing gp120. (**A**) Volcano plot of differentially expressed genes, genes with significant differences in expression are shown in red (upregulated) or dark blue (downregulated). Genes with differential expression but that could not reach the significant level are shown in light red (upregulated) or light green (downregulated). Genes with no significant differential expression are shown in grey. The vertical lines represent 2.0-fold up- and down-regulation. The horizontal lines indicate an adjusted *p* value of 0.05. (Q-value = adjusted *p*-value). (**B**) Top 30 GO terms were identified in the Transcriptomics and were displayed in the biological process (green), cellular component (orange), and molecular function sections (light blue). The adjusted statistically significant values for all of the variables were negative 10-base log transformed. DEGs are abbreviation for differentially expressed genes, while GO is abbreviation for gene ontology. (**C**) The top 20 KEGG enriched gene pathway-related disorders were examined using a KEGG pathway analysis. Low q-values are highlighted in red, while high q-values are highlighted in blue; the size of the circle is proportional to the number of enriched genes. (KEGG), stands for Kyoto Encyclopedia of Genes and Genomes, (DEG) stands for Differential Expressed Genes. (**D**) Wiki Pathways enrichment analysis top 20 (*p* < 0.05).

To determine the role of gp120 in PC12 cells, RNA transcriptomics were conducted. The cells were transfected with gp120; subsequently, the RNA was extracted for the transcriptomics experiment. Figure 3A depicts the volcano plot of the upregulated and downregulated transcriptomes. In the gene ontology analysis, many genes were enriched in cell division, mitotic cell cycle, kinetochore, chromosome centriometric region, and microtubule binding (Figure 3B). In the KEGG analysis, many genes were found to be enriched in the cell cycle, cellular senescence, DNA replication, glioma, Parkinson’s disease, and steroid biosynthesis (Figure 3C). The majority of the genes were involved in cholesterol metabolism, Hedgehog signaling, Spinal cord injury, and Wnt signaling on Wiki pathway analysis (Figure 3D).

### 3.4. Proteomics Analysis of HIV-1gp120-Expressing Cells

**Figure 4:**
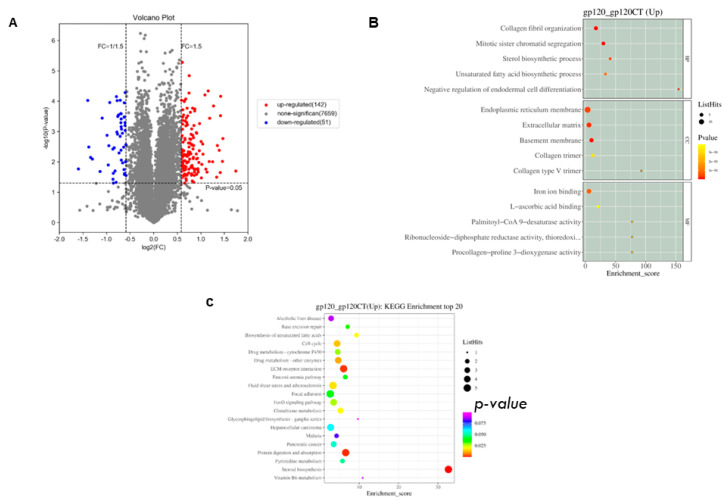
Volcano plot of DEG, GO functional analysis of DEG, and KEGG pathway enrichment analysis of upregulated genes in proteomics from PC12 cells samples overexpressing gp120. (**A**) Volcano plots showing DEGs in proteomics. Red points indicate elevated genes, blue points indicate downregulated genes, and gray points indicate no significant difference. (**B**) Top 15 GO terms were identified in the protein sequencing; q-values are highlighted in yellow; the size of the circle is proportional to the number of enriched genes. (**C**) the top 20 KEGG upregulated genes associated pathways were examined using a KEGG pathway analysis. Low q-values are highlighted in red, while high q-values are highlighted in blue to purple; the size of the circle is proportional to the number of enriched genes.

Protein analysis of the gp120-transfected PC12 cells with their corresponding controls was undertaken to identify the proteins that were activated. Our results reveal 142 upregulated and 51 downregulated proteins. Figure 4A shows the volcano plot of differentially expressed proteins. On annotation, most of the differentially expressed upregulated proteins were enriched in processes related to iron binding, sterol biosynthesis, collagen fibril organization, and biosynthesis of unsaturated fatty acid (Figure 4B). KEGG analysis of the upregulated proteins revealed that the most enriched pathways were identified in unsaturated fatty acid biosynthesis, the cell cycle, the Fanconi anemia pathway, FOXO signaling, glutathione metabolism, and steroid biosynthesis (Figure 4C).

### 3.5. Data Integration and Validation of Transcriptomics and Proteomics

**Figure 5:**
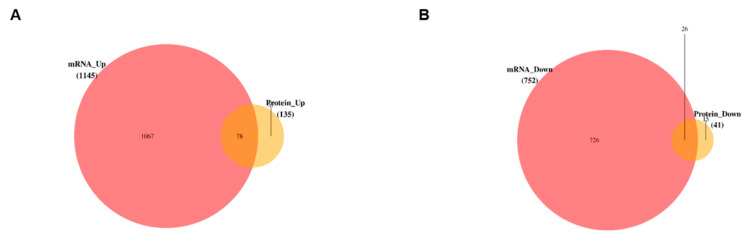

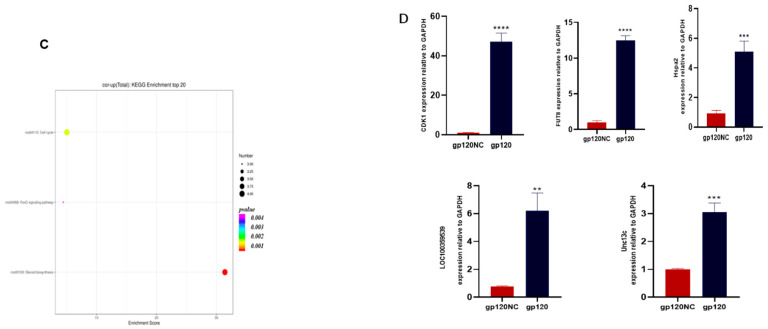
Venn diagrams showing overlaps between genes upregulated transcriptomics and proteomic analyses data sets analyses for pc12 cell lines expressing gp120. (**A**) The overlap between genes upregulated in transcriptomics and proteomics (**B**) The overlap between genes downregulated in transcriptomics and proteomics data sets. (**C**) KEGG enrichment analysis of top 20 upregulated candidate genes with their respective pathways. Low q-values are highlighted in red, while high q-values are highlighted in blue to purple; the size of the circle is proportional to the number of enriched genes. Candidate genes are those genes overlapped between in transcriptomics and proteomics. (**D**) Significant upregulation of candidate genes in PC12 by gp120 in qPCR, n = 3. *t*-test ** *p* < 0.01, *** *p* < 0.001, **** *p* < 0.0001.

To establish and validate the molecular mechanism of gp120 action in PC12 cells, the transcriptomics and proteomics data were integrated, and revealed 78 overlapping upregulated genes and 26 overlapping downregulated genes (Figure 5A,B). Next, we validated five genes through a quantitative polymerase chain reaction, Table 1 contains a list of these genes. The results indicate the significant expression of *Fut8*, *Unc13c*, *Cdk1*, *LOC100359539*, and *Hspa2* in the gp120-transfected cells, relative to the control.

### 3.6. CDK1 Is Differentially Upregulated in HIV-Associated Dementia

**Figure 6:**
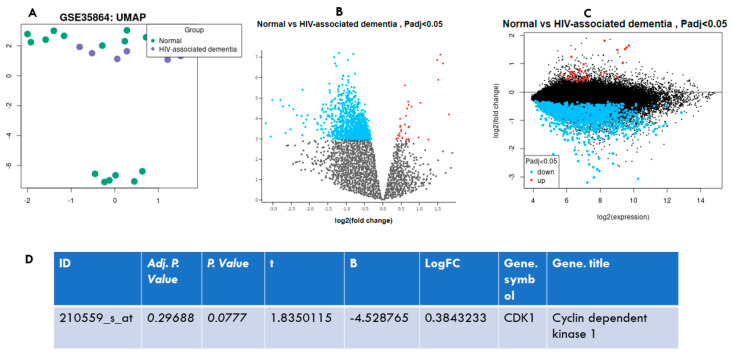
*CDK1* in HIV-associated dementia. (**A**) The Uniform Manifold Approximation and Projection plot (UMAP), (**B**) Volcano plot, and (**C**) Mean-difference plot. (**D**) indicates the *p*-value and the Log2FC of *CDK1* in HIV-associated dementia against the normal control.

In order to ascertain the glimmer involvement of *CDK1* in patients with neurological impairment secondary to HIV, we performed bioinformatics analysis. The UMAP plot indicates a cluster of both groups; unexpectedly, however, two clusters were found in a normal control group (Figure 6A). The upregulated and downregulated genes (padj ˂ 0.05) are denoted by red and blue color, consecutively (Figure 6B,C). Figure 6D indicate the *p*-value and Log2FC of *CDK1*.

### 3.7. A Pharmacological Inhibition of Cdk1 May Attenuate the gp120 Overexpression-Induced Cell Death

**Figure 7:**
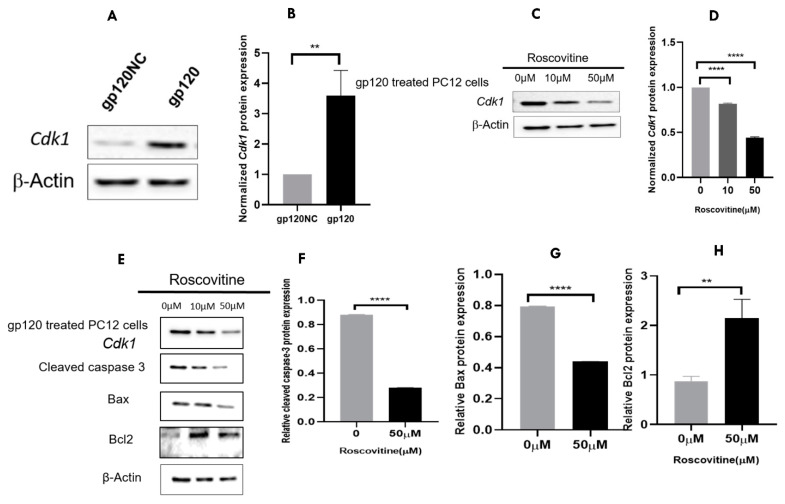
*Cdk1* gene expression before and after treatment with a Roscovitine inhibitor. (**A**) Using Western blotting, the *Cdk1* protein expression in PC12 cells expressing gp120 was compared to that in untreated cells. (**B**) The gp120 treatment group showed a significant increase in CDK1 protein expression than the untreated group. (**C**) Western blotting shows that Roscovitine inhibits CDK1 protein expression in gp120-expressing cells. (**D**) After 12 h, the administration of Roscovitine dramatically decreased CDK1 expression at the concentration of 50 µM. (**E**–**H**) Apoptosis-related proteins expression (Cleaved caspase-3, Bax, and Bcl2) in PC12- cells expressing gp120 treated with inhibitor as compared to untreated cells. Each Western blotting was loaded with control, B-Actin, n = 3. *t*-test, ** *p* < 0.01, ordinary one-way ANOVA **** *p* < 0.0001.

In order to explore the expression of CDK1 in gp120-expressing cells, we performed Western blotting analysis. The CDK1 protein expression level was higher in gp120-expressing cells than the cells transfected with normal control. Subsequently, Roscovitine (CDK1 inhibitor) was used to inhibit the expression of *Cdk1*. The results indicate that a group treated with 50 µM of Roscovitine showed a significant decrease in CDK1 in the gp120 transfected cells (Figure 7A–D). Similarly, when we examined the expression of apoptosis-related markers, we found that the inhibition of *Cdk1* by Roscovitine affected cleavage caspase 3 and BAX (decreased significantly at 50 µM), while BCL2 increased moderately (Figure 7E–H).

## 4. Discussion

Despite advances in HIV-1 management, HAND constitutes a persistent complication. Glycoprotein 120 is one of the HIV-1 neurotoxins associated with neurodegeneration, although the mechanism of this neuropathology remains perplexing. Thus, it is vital to identify the possible mechanisms of gp120-associated neuronal death. In this study, we first established that gp120 expression in PC12 induces cell death and apoptosis. Our results are concordant with the previous findings, showing that gp120 might induce apoptosis. Additionally, it has been documented that gp120 triggers neuronal death through the activation of caspase-3 and NMDAR [25,26]. However, the present study identified previously unreported genes that are likely linked to gp120-associated cell death and apoptosis. The gene ontology and KEGG enrichment analysis of the top 20 differentially overlapping upregulated genes reveal that the preponderances are involved in cell division. Cell-cycle dysregulation induces neuronal cell death and apoptosis [27]. Previous research has established a relationship between cell-cycle events and neuronal cell death [28]. Furthermore, HIV-1 dysregulates the cell cycle and generates cells that produce viral envelopes, which are linked with contagious apoptosis that spreads to other uninfected cells [29]. However, the underlying molecular mechanism is unknown. Our study revealed that gp120 protein upregulates cell-cycle signaling and induces cell death and apoptosis. This effect of gp120 may constitute the potential pathogenic mechanism of HAND. Finally, we identified the overlapping upregulated genes in RNA and protein sequencing as *Fut8*, *Unc13c*, *Cdk1*, *Loc100359539*, and *Hspa2*. However, Cyclin-dependent kinase 1(*Cdk1*) was the most upregulated gene. *Cdk1* is a key protein kinase that directs cells into normal mitosis [30,31], *its* upregulation promotes cell death and apoptosis [32,33]. Our findings show that gp120 upregulated *Cdk1* and induced cellular apoptosis. More importantly, in vivo and vitro HIV-1 clinical experiments demonstrated the unique existence of a *Cdk1*-linked proapoptotic pathway [20]. To support the involvement of cyclins in HIV proteins, it has been reported that HIV-1 Tat increases the calpain-1 cleavage of p35 to p25 via calcium dysregulation, which hyper activates CDK5, resulting in the aberrant phosphorylation of downstream targets such as Tau, CRMP2, DCX, and MEF2. Moreover, Tat disrupts CDK5’s nuclear-cytoplasmic transport, which eventually leads to a buildup of aberrantly phosphorylated cytoplasmic targets [Tau, CRMP2, DCX], and ultimately impairs the neuronal function and, hence, leads to cell death [34]. The aberrant activation of *Cdk1* is thought to be involved in apoptosis associated with HIV-1 infection and neurodegenerative illness [35]. Cdk1, mTOR, and p53 play sequential roles in apoptosis caused by the HIV-1 envelope [36].

The present work is also supported by bioinformatics analysis, which we performed on postmortem brain samples from individuals with HIV-associated dementia and compared to the normal control. This analysis proved that *CDK1* is upregulated in the HIV-associated dementia group (which is linked with the expression of gp120 protein) compared to the normal control. Our work provides further evidence that gp120 upregulates *Cdk1*, a mechanism that may trigger apoptosis and cell death. We also demonstrate that the pharmacological targeting of *Cdk1* signaling in cells that express gp120 may protect neurons. Roscovitine, a selective *Cdk* inhibitor that targets *Cdk1*, reduces neuronal death in accordance with this [37] Roscovitine and numerous other small chemical inhibitors of CDKs have been widely researched, having been utilized in both in vitro and in vivo models of neuroprotection [37,38]. Our findings show that 50 µM of Roscovitine suppresses *Cdk1*, which could be an alternative way to protect neurons from gp120-induced death.

## 5. Conclusions

Despite decades of HIV-1 studies that have yielded important advances in infection management and treatment, there remains a long fight against this virus. HAND has become more contagious, and HIV-1 viral proteins, specifically gp120, are potentially associated with neurodegeneration, although the exact mechanism remains unclear. Our research revealed that gp120 causes apoptosis and cell death and upregulates the cell-cycle pathway, suggesting a potential relationship to neurodegeneration. Furthermore, we discovered *Cdk1* as the key upregulated gene associated with cell death and apoptosis. This was validated through analysis of postmortem brain samples and the pharmacological inhibition of *Cdk1*. This study provides insight into the molecular mechanisms associated with gp120-associated neuronal apoptosis and death, as the hallmarks of HAND. This could facilitate the diagnosis and development of therapeutic targets to help combat the disease.

## Figures and Tables

**Table 1:** Primers used for qPCR.

GENE	Sequences	Product Size (bp)
*HIV-1gp120* F	TAGAGCTAGCGAATTCATGAGAGTGACGGGGATCAGG	37
*HIV-1gp120* R	CACCTCCACCGGATCCGCGCTTTTCTCTCTCCACC	35
*Fut8* F	ACGTGGTTCGTTGACAGACA	20
*Fut8* R	TACTGTGCATGGGCTTGAGG	20
*Unc13c* F	GACTGCCTACACCCCTGTTC	20
*Unc13c* R	CCCGCAGTTGTTGGATGTTG	20
*Cdk1* F	CCGGTTGACATCTGGAGCAT	20
*Cdk1* R	TAAACGCCACGATCTTCCCC	20
*LOC100359539* F	GCAGCCCCATTCAGAGTCTT	20
*LOC100359539* R	GTGAACTACCCCAGGGAACG	20
*Hspa2* F	CCATCGCTTATGGCCTGGAT	20
Hspa2 R	TGCTGGAGGGATCCCAGTTA	20
Gapdh F	CGTCCCGTAGACAAAATGGTGAA	23
*Gapdh* R	GCCGTGAGTGGAGTCATACTGGAACA	26

## Supplementary Materials

The following are available online at https://www.mdpi.com/article/10.3390/v14122793/s1, Supplementary File S1: The detailed protocol for RNA extraction and all transcriptomics; Supplementary File S2: The detailed protocol for protein extraction and proteomics.

## Abbreviations

AIDS—acquired immune deficiency syndrome, CCK8—Cell Counting Kit 8, CNS—Central Nervous System, DEGS—Differential Expressed Genes, GFP—Green Fluorescence Plasmid, GO—Gene ontology, gp120—glycoprotein 120, HAND—HIV associated neurocognitive disorder, HIV-1—Human immunodeficiency virus-1, KEGG—Kyoto Encyclopedia of Genes and Genomes, NMDAR—N-Methyl-D-aspartate receptor, PEI—polyethylenimine.
