# Supplementary material for: HIV-1 gp120 Protein Activates Cyclin-Dependent Kinase 1, a Possible Link to Central Nervous System Cell Death
Source: Viruses. 2022 Dec 15;14(12):2793. doi: 10.3390/v14122793 (PMC9786227; doi:10.3390/v14122793)
Supplement: Supplementary file 1 [file viruses-14-02793-s001.zip › Supplementary S1.pdf]

Transcriptomics

RNA Isolation and Library Preparation

Total RNA was extracted using the TRIzol reagent according to the manufacturer's protocol. RNA purity and quantification were evaluated using the NanoDrop 2000 spectrophotometer (Thermo Scientific, USA). RNA integrity was assessed using the Agilent 2100 Bioanalyzer (Agilent Technologies, Santa Clara, CA, USA). Then the libraries were constructed using TruSeq Stranded mRNA LT Sample Prep Kit (Illumina, San Diego, CA, USA) according to the manufacturer's instructions. The transcriptome sequencing and analysis were conducted by OE Biotech Co., Ltd. (Shanghai, China).

RNA Sequencing and Differentially Expressed Genes Analysis

The libraries were sequenced on an Illumina HiSeq X Ten platform and 150 bp paired-end reads were generated. About xxxxx raw reads for each sample were generated. Raw data (raw reads) of fastq format were firstly processed using Trimmomatic [1] and the low quality reads were removed to obtain the clean reads. Then about xxxxxx clean reads for each sample were retained for subsequent analyses.

The clean reads were mapped to the human genome (GRCh38) using HISAT2 [2]. FPKM [3] of each gene was calculated using Cufflinks [4], and the read counts of each gene were obtained by HTSeq-count [5]. Differential expression analysis was performed using the DESeq (2012) R package [6]. P value < 0.05 and fold change > 2 or fold change < 0.5 was set as the threshold for significantly differential expression. Hierarchical cluster analysis of differentially expressed genes (DEGs) was performed to demonstrate the expression pattern of genes in different groups and samples. GO enrichment and KEGG [7] pathway enrichment analysis of DEGs were performed respectively using R based on the hypergeometric distribution.

After reads reassembled by StringTie [8], gene structure extension and novel transcripts identification were performed by comparing the reference genome and the known annotated genes using Cuff compare software. AS profile [9] was used to analyse the alternatively splicing of differentially regulated transcripts isoforms or exons. SNPs and In Dels were called using SAM

tools [10] and BCF tools [11], and the details were shown on SAM tools webpage (http://samtools.sourceforge.net/mpileup.shtml). Then SnpEff [12] annotates and predicts the effects of variants on genes (such as amino acid changes).

Reference:

1 Bolger AM, Lohse M & Usadel B. Trimmomatic: a flexible trimmer for Illumina sequence data. Bioinformatics 2014; 30(15): 2114-2120.

2 Kim D, Langmead B & Salzberg SL. HISAT: a fast spliced aligner with low memory requirements. *Nature Methods* 2015; 12(4): 357-360.

3 Roberts A, Trapnell C, Donaghey J, *et al*. Improving RNA-Seq expression estimates by correcting for fragment bias. *Genome Biology* 2011; 12(3): R22.

4 Trapnell C, William BA, Pertea G, *et al*. Transcript assembly and quantification by RNA-Seq reveals unannotated transcripts and isoform switching during cell differentiation. *Nature Biotechnolgy* 2010; 28(5): 511-515.

5 Anders S, Pyl PT & Huber W. HTSeq--a Python framework to work with high-throughput sequencing data. *Bioinformatics* 2015; 31(2): 166-169.

6 Anders S & Huber W. Differential expression of RNA-Seq data at the gene level - the DESeq package. EMBL, 2013.

7 Kanehisa M, Araki M, Goto S, *et al*. KEGG for linking genomes to life and the environment. *Nucleic Acids Research* 2008; 36: D480-D484.

8 Pertea M, Pertea GM, Antonescu CM, *et al*. StringTie enables improved reconstruction of a transcriptome from RNA-seq reads. *Nature Biotechnology* 2015; 33(3): 290-295.

9 Florea L, Song L & Salzberg SL. Thousands of exon skipping events differentiate among splicing patterns in sixteen human tissues. *F1000Research* 2013; 2:188.

10 Li H, Handsaker B, Wysoker A, *et al*. The Sequence Alignment/Map (SAM) Format and SAM tools. *Transplantation Proceedings* 2009; 19(1 Pt 2):1653-1654.

11 Li H. A statistical framework for SNP calling, mutation discovery, association mapping and population genetical parameter estimation from sequencing data. *Bioinformatics* 2011; 27(21): 2987-2993.

12 Cingolani P, Platts A, Wang le L, *et al.* A program for annotating and predicting the effects of single nucleotide polymorphisms, SnpEff: SNPs in the genome of Drosophila melanogaster strain w1118; iso-2; iso-3. *Fly* 2012; 6(2): 80-92.
