# Supplementary material for: HIV-1 gp120 Protein Activates Cyclin-Dependent Kinase 1, a Possible Link to Central Nervous System Cell Death
Source: Viruses. 2022 Dec 15;14(12):2793. doi: 10.3390/v14122793 (PMC9786227; doi:10.3390/v14122793)
Supplement: Supplementary file 1 [file viruses-14-02793-s001.zip › Supplementary S2.pdf]

Proteomics

Chapter 1 Sample Pretreatment

1.1 Ultrasonic cracking

Frozen samples were transferred into low protein binding tubes (1.5ml Eppendorf) and lysed with 300 μL lysis buffer supplemented with 1mM PMSF. Then samples were further lysed with sonication. The parameters were set as 1s/1s intervals, 3min time and 80W power. After sonication, the samples were centrifuged at 15000 g for 15 min to remove insoluble particles, repeat once to further exclude precipitation. Protein concentration was determined by BCA assay and aliquoted to store at -80°C.

1.2 Sonication breaks acetone precipitation

Frozen samples were transferred into low protein binding tubes (1.5ml Eppendorf) and lysed with 300 μL lysis buffer supplemented with 1mM PMSF. Then the samples were further lysed with sonication. The parameters were set as 1s/1s intervals, 3min time and 80W power. After sonication, the samples were centrifuged at 15000 g for 15 min to remove insoluble particles, repeat once to further exclude precipitation. The supernatants were added 5 times the volumes of cold acetone and precipitated at -20°C overnight and centrifuged at 12000g for 10min at 4°C to collect precipitation. The precipitation were dried at room temperature for 3min and dissolved in SDS lysis buffer for 2hrs. Furthermore, the samples were centrifuged at 12000g for 10min to collect supernatants and centrifuged again to remove precipitation completely. Protein concentration was determined by BCA assay and aliquoted to store at -80°C.

1.3 Phenol extraction

Frozen samples were transferred into low protein binding tubes (2ml Eppendorf). The 500 μL extraction buffer was added to each sample with steel beads and the samples were grinded at the power of 60Hz for 2min. The samples were then supplemented with extraction buffer to 1ml and mixed and the mixtures were added with Tris-phenol buffer and mixed for 30min at 4°C. Furthermore, the mixtures were centrifuged at 7100g for 10min at 4°C to collect phenol supernatants. The supernatants were added for 5 times the volumes of 0.1M cold ammonium acetate -methanol buffer and precipitated at -20°C overnight. After precipitation, the samples were centrifuged at 12000g for 10min to collect precipitation. Then the precipitation were washed by 5 times the volumes of cold methanol and gently mixed. The precipitation were centrifuged at 12000g for 10min at 4°C again to collect precipitation and repeat once. Then methanol was replaced by acetone and repeat wash step twice to remove methanol contamination. Furthermore, the samples were centrifuged at 12000 g for 10 min at 4°C to collect precipitation and the precipitation were dried at room Temperature for 3min and dissolved in lysis buffer for 3hrs. Finally, the samples were centrifuged at 12000g for 10min to collect supernatants. The supernatants were centrifuged again to remove precipitation completely. Protein concentration was determined by BCA assay and aliquoted to store at -80°C.

1.4 Serum protein extraction method

1. Equilibrate the depletion spin column to room temperature.
2. Remove the column screw cap and add 10μL of sample directly to the resin slurry in the column.
3. Cap the column and invert the column several times until the resin is completely suspended in the solution.
4. Incubate the mixture in the column with gentle end-over-end mixing for 60 minutes at room temperature. Make sure the sample mixes with the resin during the incubation period. Alternatively, vortex every 5 minutes.
5. Twist off the bottom closure and loosen the cap. Place column into a 2mL collection tube and centrifuge at 1000 × g for 2 minutes.
6. Discard the column containing the resin.
7. Filtrate contains sample with the top 12 proteins removed. Use for further processing or store for later use. The depleted sample will be in
10mM PBS, 0.15M NaCl, 0.02% azide, pH 7.4.

1.5 Homogenization ultrasonic method

Frozen samples were transferred into low protein binding tubes (1.5ml Eppendorf) and lysed with 500 μL digestion buffer supplemented with 1mM PMSF. Then the tissue samples were homogenized on the ice and further lysed with sonication. The parameters were set as 1s/1s intervals, 3min time and 80W power. After sonication, the samples were centrifuged at 15000 g for 15 min at 4°C to remove insoluble particles, repeat once to further exclude precipitation. Protein concentration was determined by BCA assay and aliquoted to store at -80° C.

1.6 Determination of protein concentration

1. BCA

Quantitative Principle

BCA (bicinchonininc acid) is mixed with other reagents such as copper sulfate of divalent copper ions to form apple green, which is BCA working reagent. Under alkaline conditions, when BCA binds to protein, the protein reduces Cu^{2+} to Cu^{+}, and one Cu^{+} chelates two BCA molecules. The working reagent forms a purple complex from the original apple green. The water-soluble complex exhibits the maximum absorbance at 562 nm. The absorbance has a good linear relationship with protein concentration in a wide range. Therefore, the protein concentration can be calculated according to the absorbance value.

BCA experimental process

According to the instructions of BCA kit, the required volume of BCA working reagent is allocated by buffer A: Buffer B = 50:1 (v/v). Take out part of the protein solution to be tested and dilute it with ultra-pure water (to prevent excessive concentration from exceeding the working range of the standard curve). Prepare a clean 96-well plate and add BSA standard protein solution of gradient as follows: 0,1,2,4,8,12,16,20 μL. Then add the corresponding

volume of ultra-pure water to each hole to supplement the volume to 20 μL. Two microliter protein solution was added to 96-well plate with three multiple holes for each sample, and the volume of the protein solution was added to 20 μL. Then 200 μL pre-configured working reagent was

added into each hole, and the mixture incubate for 30 minutes at the temperature of 37. The absorbance value (wavelength 562 nm) was determined by enzyme labeling instrument. Then calculating the standard curve according to the known concentration and absorbance value of the standard protein solution, and substituting the absorbance value of the sample to be measured, the protein concentration value can be calculated.

2. Bradford

Quantitative Principle

The determination of protein content by Coomassie Brilliant Blue is one of the dye binding methods. Coomassie brilliant blue is red in free state and its maximum light absorption is 488 nm. When it binds to protein, it becomes blue, and protein-pigment conjugates have maximum light absorption at 595 nm. Its light absorption value is proportional to protein content, so it can be used for quantitative determination of protein. The binding of protein with Coomassie brilliant blue reached equilibrium in about 2 minutes, and the reaction was completed quickly. The complex remained stable at room temperature for 1 hour. The reagent prepared by this method is simple, easy to operate and fast. The reaction is very sensitive. The sensitivity of this method is 4 times higher than that of Lowry method, so it can be used to determine microgram protein content, the concentration range of protein is 0-1000 μg/ml. It is a commonly used method for rapid determination of microprotein.

Bradford method experimental process

Bradford working reagent is allocated by 0.01% (w/v) G250, 8.5% phosphoric acid and 4.75% ethanol, which is preserved in a brown reagent bottle at room temperature for three months, but during this period there may be precipitation of dyes, so mix evenly before each use. Take out part of the protein solution to be tested and dilute it with ultra-pure water (to prevent excessive concentration from exceeding the working range of the standard curve). A BSA standard protein solution with concentration of 1.0 mg/ml, 0.8 mg/ml, 0.4 mg/ml, 0.2 mg/ml and 0.1 mg/ml was prepared. The 96-well plate was added with 10 μL BSA standard protein solution per hole, and then 90 μL water was added to supplement the volume to 100 μL. Two microliter protein solution was added to 96-well plate with three multiple holes for each sample, and the volume of the protein solution was added to 100 μL. Then 100 μL pre-configured working reagent was added into each hole, and the mixture was stilled for 10 minutes. The absorbance value (wavelength 595 nm) was determined by enzyme labeling instrument. Then calculating the standard curve according to the known concentration and absorbance value of the standard protein solution, and substituting the absorbance value of the sample to be measured, the protein concentration value can be calculated.

1.7 SDS-PAGE electrophoresis

The 10ug proteins of each sample were acquired and separated by 12% SDS-PAGE gel. Then separation gel was stained by CBB according to Candiano's protocol: Firstly, the gel was fixed for 2 h and stained for 12 h. After staining, the gel was washed with water until the bands were visualized. Finally, the stained gel was scanned by automatic digital gel image analysis system（Tanon 1600）.

Chapter II: Protease and Labeling

2.1 Protease

2.1.1 FASP enzymolysis

According to the results of protein quantification, one hundred microgram protein extraction was subjected with 120 μL reducing buffer (10 MmDTT, 8 M Urea, 100 mM TEAB, pH 8.0) on 10K ultrafiltration tube. The solution was incubated at 60°C for 1 h and IAA was added to the solution with the final concentration of 50 mM in the dark for 40 min at room temperature. Then the solutions were centrifuged on the filters at 12000 rpm for 20 min at 4°C and the flow-through solution were discarded from the collection tube. One hundred microliter 300 mM TEAB was added to the solutions and centrifuged at 12000 rpm for 20 min and repeat this step twice. After washing step, the filter units were transferred into new collection tubes and 100 μL 300 mM TEAB was added and followed with 3 μl sequencing-grade trypsin (1 μg/μL) in each tube, then the solutions were incubated for digestion at 37°C for 12 h. Finally, the collections of digested peptides were centrifuged at 12000 rpm for 20 min. 50μL 200 mM TEAB were added and centrifuged again. The solutions were collected and lyophilized.

2.1.2 Enzymatic digestion in solution

According to the measured protein concentration, take the same quality protein from each sample, and dilute different groups of samples to the same concentration and volume. Add 25mm DTT of corresponding volume into the above protein solution to make the DTT final Concentration about 5mM, and incubate at 55°C for 30-60min. Then add the corresponding volume of iodoacetamide so that the final concentration is about 10mm, and place in the dark for 15-30min at room temperature. Then 6 times of the volume of precooled acetone in the above system to precipitate the protein, and place it at - 20 °C for more than four hours or overnight. After precipitation, take out the sample and centrifuge at 8000g for 10 min at 4 °C for collecting the precipitate. According to the amount of protein, add the corresponding volume of enzymolysis diluent (protein: enzyme = 50:1 (m/m), 100ug of protein add 2ug of enzyme) to dissolve the protein precipitate, then the solutions were incubated for digestion at 37°C for 12 h. Finally, samples were lyophilized or evaporated after enzymolysis.

2.2 Peptide labeling

2.2.1 iTRAQ mark

For iTRAQ labelling, the lyophilized samples were suspended in 100 μL 200 mM TEAB and 40 μL of each sample were transferred into new tubes for labeling. Two hundred microliter isopropanol was added to iTRAQ reagent vial at room temperature. The centrifuged reagents were dissolved for 5 min and mixed for centrifugation and repeat this step once. Then100 μL of the iTRAQ label reagent was added to each sample for mixing. The tubes were incubated at room temperature for 2 h. Finally, 200 μL of HPLC water was added to each sample and incubated for 30 min to terminate reaction. The labeling peptides solutions were lyophilized and stored at -80°C.

2.2.2 TMTMark (6 and 10 bids)

For TMT labelling, the lyophilized samples were resuspended in 100 μL 200 mM TEAB and 40 μL of each sample were transferred into new tubes for labeling. Eighty-eight microliter acetonitrile were added to TMT reagent vial at room temperature. The centrifuged reagents were dissolved for 5 min and mixed for centrifugation and repeat this step once. Then 41 μL of the TMT label reagent was added to each sample for mixing. The tubes were incubated at room temperature for 1 h. Finally, 8 μL of 5% hydroxylamine were added to each sample and incubated for 15 min to terminate reaction. The labeling peptides solutions were lyophilized and stored at -80°C.

2.2.3 TMT mark (16 standards)

For TMTpro 16 labelling, the lyophilized samples were resuspended in 100 μL 100 mM TEAB pH8.5 and 40 μL of each sample were transferred into new tubes for labeling. Anhydrous acetonitrile were added to TMT reagent vial at room temperature. The centrifuged reagents were dissolved for 5 min and mixed for centrifugation and repeat this step once. Then 10 μL of the TMTpro label reagent was added to each sample for mixing. The tubes were incubated at room temperature for 1 h. Finally, 5 μL of 5% hydroxylamine were added to each sample and incubated for 15 min to quench the reaction. The labeling peptides solutions were lyophilized and stored at -80°C.

2.3Desalting

2.3.1 Waters Large pillars

The solutions were added 2-4μL H3PO4 to each sample to adjust the pH value to 1-3.The digested peptides were desalted by C18-Reverse-Phase SPE Column. Firstly, the column was washed by 1mL methanol twice, followed by 0.1%TFA/H2O 2-3 times. And the samples were loaded on the column 3 times. Then the column was washed by 0.1%TFA/H2O 3 times. Finally, the peptides was eluted with 90% ACN/H2O (containing 0.1%TFA) 3 times and were lyophilized.

2.3.2 SOLA™ SPE 96Orifice plate

The solutions were added about 1μL H_3PO_4 to each sample to adjust the pH value to 7.The digested peptides were desalted by SOLA™ SPE 96Orifice plate Column. Firstly, the column was washed by 200 μ L methanol 3 times, followed by 200μL H_2O 2-3 times. And the samples were loaded on the column twice. Then the column was washed by 5% methanol/H_2O 3 times. Finally, the peptides was eluted with 150μL 100% methanol 3 times and were lyophilized.

2.4 Phosphorylation enrichment

Phosphopeptides were enriched from samples using titanium dioxide beads (TiO_2) according to a modified protocol from Jersie-Christensen et al.[3].

1) Centrifuge the sample tubes for 3 minutes at room temperature, 12000 rpm to precipitate peptides to the bottom of the tube.
2) Resolve the phosphopeptides into enrichment kit loading buffer and vortex for 15 min.
3) Briefly centrifuge to collect the fluid on the wall.
4) Add TiO_2 beads (protein: TiO_2=1:4, m/m) resolution and vortex for 15 min.
5) Centrifuge for 1 min at 6000 rpm, RT, collect the sediment.
6) Add 400 μL wash buffer 1 (0.5% TFA/ 50% ACN) to the sediment, vortex for 15min; then centrifuge for 1 min at room temperature, 6000 rpm, collect the sediment. Repeat one more time.
7) Add 400 μL wash buffer 2 (0.1% TFA/ 50% ACN) to the sediment, vortex for 15min; then centrifuge for 1 min at room temperature, 6000 rpm, collect the sediment. Repeat one more time.
8) Add 100 μL 10% $NH_3 \cdot H_2O$ to wash off the phosphor-peptides, vortex for 15min; then centrifuge for 3 min at room temperature, 8000 rpm, collect the supernatant and repeat one more time.
9) Collect all supernatant, then centrifuge for 5 min at room temperature, 12000 rpm and collect the supernatant.
10) After the steps above, the supernatant is the final enriched phosphor-peptides solution, which can be vacuum concentrated for further use

3.1 AB5600 Chromatographic conditions versus mass spectrometry conditions (labeling)

RPLC analysis.

RP separation was performed on an 1100 HPLC System (Agilent) using an Agilent Zorbax Extend RP column (5 μm, 150 mm × 2.1 mm). Mobile phases A (2% acetonitrile in HPLC water) and B (98% acetonitrile in HPLC water) were used for RP gradient. The solvent gradient was set as follows: 0~8 min, 8% A; 8~8.01 min, 98%~95% A; 8.01~48 min, 95%~75% A; 48~60 min, 75~60% A; 60~60.01 min, 60~10% A;60.01~70 min, 10% A; 70~70.01 min, 10~98% A; 70.01~75 min, 98% A. Tryptic peptides were separated at an fluent flow rate of 300μL/min and monitored at 210 and 280 nm. Samples were collected

for 8-60 minutes, and eluent was collected in centrifugal tube 1-15 every minute in turn. Samples were recycled in this order until the end of gradient. The separated peptides were lyophilized for mass spectrometry.

Mass spectrometry analysis

All analyses were performed by a Triple TOF 5600 mass spectrometer (SCIEX, USA) equipped with a Nanospray III source (SCIEX, USA). Samples were loaded by a capillary C18 trap column (3 cm × 100 μm) and then separated by a C18 column (15 cm × 75 μm) on an E ksigentnanoLC-1D plus system (SCIEX, USA). The flow rate was 300 nL/min and linear gradient was set as follows: 0~0.5 min, 95%~92% A; 0.5~48 min, 92%~74% A; 48~61 min, 74%~62% A; 61~61.1 min, 62%~15% A; 61.1~67 min, 15% A; 67~67.1 min, 15%~95% A; 67.1~70 min, 95% A. Mobile phase A = 2% ACN/0.1% FA and B = 95% ACN/0.1% FA.Data were acquired with a 2.4 kV ion spray voltage, 35 psi curtain gas, 5 psi nebulizer gas, and an interface heater temperature of 150° C. The MS scanned between 400 and 1500 with an accumulation time of 250 ms. For IDA, 30 MS/MS spectra (80 ms each, mass range 100 – 1500) of MS peaks above intensity 260 and having a charge state of between 2 and 5 were acquired. A rolling collision energy voltage was used for CID fragmentation for MS/MS spectra acquisitions. Mass was dynamically excluded for 22 seconds.

Database search

ProteinPilot software (v.5.0) was used to search all of the TripleTOF 5600 MS/MS raw data thoroughly against the sample protein database. Database search was performed with Trypsin digestion specificity, and the cysteine alkylation was considered as parameters in the database searching. For protein quantification method, labeling method (2.1 Protease) was selected. A global false discovery rate (FDR) of <1% was used and peptide groups considered for quantification required at least 2 peptides.

3.2 AB6600 Chromatographic conditions versus mass spectrometry conditions (labeling)

NanoLC-ESI-MS/MS analysis

The lyophilized peptide fractions were re-suspended in 2% acetonitrile containing 0.1% formic acid, and 4ul aliquots of which was loaded into a ChromXP C18 (3 μm, 150 Å) trap column. The online Chromatography separation was performed on the Ekspert nanoLC 415 system (SCIEX, Concord, ON), The trapping, desalting procedure were carried out at a flow rate of 4μL/min for 5 min with 100% solvent A (water/acetonitrile/formic acid = 98/2/0.1% (v/v/v). Composition of solvent B was water/acetonitrile/formic acid = 5/95/0.1% (v/v/v). Then, an elution gradient of 5-85% solvent B over 67 min (followed by 95% A over 3 min) was used on an analytical column (75 μm x 15 cm C18- 3μm 120 Å, ChromXP, Eksigent). IDA (information-dependent acquisition) mass spectrum techniques were used

to acquire tandem MS data on a Triple TOF 6600 tandem mass spectrometer (Sciex, Concord, Ontario, Canada) fitted with a Nanospray III ion source. Data was acquired using an ion spray voltage of 2.4 kV, curtain gas of 35 PSI, nebulizer gas of 12 PSI, and an interface heater temperature of 150℃. The MS was operated with TOF-MS scans. For IDA, survey scans were acquired in 250 ms and up to 40 product ion scans (50ms) were collected if exceeding a threshold of 260 cps with a charge state of 2-4. A Rolling collision energy setting was applied to all precursor ions for collision-induced dissociation. Dynamic Exclusion was set for 16 s.

Database searching and protein quantification

The MS/MS data were analyzed for protein identification and quantification using Protein Pilot software (v.5.0). The local false discovery rate was estimated with the integrated PSPEP tool in the ProteinPilot Software to be 1.0% after searching against a decoy concatenated uniprot XX protein database. The following settings were selected: Sample type: Identification; Cys Alkylation: Iodoacetamide; Digestion: Trypsin; Instrument: TripleTOF 6600; Special Factors: Urea denaturation; Species: None; Search effort: Thorough ID. Only proteins identified at global FDR ⩽ 1% and unique peptides ⩾ 1 were considered for protein lists and for further downstream analysis. For a protein to be determined as deferentially expressed, it must have been identified and quantified with at least 1 significant peptide and the p-values of the protein's quantitation should be less than 0.05 and fold change ⩾ 1.5.

3.3 label free

Liquid chromatography-mass spectrometry All analyses were performed by a QE mass spectrometer (Thermo, USA) equipped with an Easy spray source (Thermo, USA). Samples were loaded by a capillary trap column (100μm×2cm, RP-C18, Thermo Fisher) and then separated by a capillary analytical column (15 cm × 75 μm, RP-C18, Thermo Fisher) on an EASY-nLCTM 1200 system (Thermo, USA). The flow rate was 300 nL/min and linear gradient was set as follow: 0~40 min，5-30% B；40~55 min，30-50% B；55~60min，50-100% B.Full MS scans were acquired in the mass range of 300-1600 m/z with a mass resolution of 70000 and the AGC target value was set at 1e6.The 10 most intense peaks in MS were fragmented with higher-energy collisional dissociation (HCD) with collision energy of 28. MS/MS spectra were obtained with a resolution of 35000 with an AGC target of 2e5 and a max injection time of 80 ms. The dynamic exclusion was set for 15.0 s and run under positive mode.

Database search

The LC-MS/MS raw data were imported in Maxquant for labeling free quantification analysis and the search engine was Andromeda. The database was offered by researchers. For limiting a certain number of peak matches by chance a target decoy- based false

discovery rate (FDR) approach is utilized. For peptide identification, mass and intensity of the peptide peaks in a mass spectrometry (MS) spectra are detected and assembled into three-dimensional (3D) peak hills over the m/z retention time plane, which are filtered by applying graph theory algorithms to identify isotope patterns. High mass accuracy is achieved by weighted averaging and through mass recalibration by subtracting the determined systematic mass error from the measured mass of each MS isotope pattern. Peptide and fragment masses (in case of an MS/MS spectra) are searched in an organism specific sequence database, and are then scored by a probability-based approach termed peptide score. The assembly of peptide hits into protein hits to identify proteins is the next step. The assembly of peptide hits into protein hits to identify proteins is the next step, in which each identified peptide of a protein contributes to the overall identification accuracy. The organism specific database search includes not only the target sequences, but also their reverse counterparts and contaminants, which helps to determine a statistical cutoff for acceptable spectral matches.

3.4 QE Chromatographic conditions versus mass spectrometry conditions (labeling)

RPLC analysis RP separation was performed on an 1100 HPLC System (Agilent) using an Agilent Zorbax Extend RP column (5 μm, 150 mm × 2.1 mm).Mobile phases A (2% acetonitrile in HPLC water) and B (98% acetonitrile in HPLC water) were used for RP gradient. The solvent gradient was set as follows: 0~8 min, 8% A; 8~8.01 min, 98%~95% A; 8.01~48 min, 95%~75% A; 48~60 min, 75~60% A; 60~60.01 min, 60~10% A;60.01~70 min, 10% A; 70~70.01 min, 10~98% A; 70.01~75 min, 98% A. Tryptic peptides were separated at an fluent flow rate of 300μL/min and monitored at 210 and 280 nm. Samples were collected for 8-60 minutes, and eluent was collected in centrifugal tube 1-15 every minute in turn. Samples were recycled in this order until the end of gradient. The separated peptides were lyophilized for mass spectrometry.

Mass spectrometry analysis

TMT 6

All analyses were performed by a Q-Exactive mass spectrometer (Thermo, USA) equipped with a Nanospray Flex source (Thermo, USA). Samples were loaded and separated by a C18 column (15 cm × 75 μm) on an EASY-nLCTM 1200 system (Thermo, USA). The flow rate was 300nL/min and linear gradient was 60 min (0~40 min, 5-30% B; 40~54 min, 30-50% B; 54~55 min, 50-100% B; 55~60 min, 100%B; mobile phaseA = 0.1% FA in water and B = 80% ACN/0.1% FA in water).Full MS scans were acquired in the mass range of 300 – 1600 m/z with a mass resolution of 70000 and the AGC target value was set at 1e6.The ten most intense peaks in MS were fragmented with higher-energy collisional dissociation (HCD) with NCE of 32. MS/MS spectra were obtained with a resolution of 17500 with an AGC target of 2e5 and a max injection time of 80 ms. The Q-E dynamic exclusion was set for 30.0 sand run under positive mode. MT 10

All analyses were performed by a Q-Exactive mass spectrometer (Thermo, USA) equipped with a Nanospray Flex source (Thermo, USA).Samples were loaded and separated by a C18 column (15 cm × 75 μm) on an EASY-nLCTM 1200 system (Thermo, USA). The flow rate was 300nL/min and linear gradient was 60 min (0~40 min, 5-30% B; 40~54 min, 30-50% B; 54~55 min, 50-100% B; 55~60 min, 100%B; mobile phaseA = 0.1% FA in water and B = 80% ACN/0.1% FA in water).Full MS scans were acquired in the mass range of 300 – 1600 m/z with a mass resolution of 70000 and the AGC target value was set at 1e6.The ten most intense peaks in MS were fragmented with higher-energy collisional dissociation (HCD) with NCE of 32. MS/MS spectra were obtained with a resolution of 17500 with an AGC target of 2e5 and a max injection time of 80 ms. The Q-E dynamic exclusion was set for 30.0 s and run under positive mode. iTRAQ All analyses were performed by a Q-Exactive mass spectrometer (Thermo, USA) equipped with a Nanospray Flex source (Thermo, USA). Samples were loaded and separated by a C18 column (15 cm × 75 μm) on an EASY-nLCTM 1200 system (Thermo, USA). The flow rate was 300nL/min and linear gradient was 60 min (0~40 min, 5-30% B; 40~54 min, 30-50% B; 54~55 min, 50-100% B; 55~60 min, 100%B; mobile phase A = 0.1% FA in water and B = 80% ACN/0.1% FA in water).Full MS scans were acquired in the mass range of 300 – 1600 m/z with a mass resolution of 35000 and the AGC target value was set at 1e6. The ten most intense peaks in MS were fragmented with higher-energy collisional dissociation (HCD) with NCE of 30. MS/MS spectra were obtained with a resolution of 17500 with an AGC target of 2e5 and a max injection time of 80ms. The Q-E dynamic exclusion was set for 30.0 s and run under positive mode.

Database search

Proteome Discoverer (v.2.4) was used to search all of the Q Exactive raw data thoroughly against the sample protein database. Database search was performed with Trypsin digestion specificity. Alkylation on cysteine was considered as fixed modifications in the database searching. For protein quantification method, labeling method was selected. A global false discovery rate (FDR) was set to 0.01 and protein groups considered for quantification required at least 2 peptides.

3.5 QE HF Chromatographic conditions versus mass spectrometry conditions (labeling)RPLC analysis

RP separation was performed on an 1100 HPLC System (Agilent) using an Agilent Zorbax Extend RP column (5 μm, 150 mm × 2.1 mm).Mobile phases A (2% acetonitrile in HPLC water) and B (98% acetonitrile in HPLC water) were used for RP gradient. The solvent gradient was set as follows: 0~8 min, 8% A; 8~8.01 min, 98%~95% A; 8.01~48 min, 95%~75% A; 48~60 min, 75~60% A; 60~60.01 min, 60~10% A;60.01~70 min, 10% A; 70~70.01 min, 10~98% A; 70.01~75 min, 98% A. Tryptic peptides were separated at an fluent flow rate of 300μL/min and monitored at 210 and 280 nm. Samples were collected for 8-60 minutes, and eluent was collected in centrifugal tube 1-15 every minute in turn. Samples were recycled in this order until the end of gradient. The separated peptides were lyophilized for mass spectrometry. Mass spectrometry analysis TMT 6All analyses were

performed by a Q-Exactive HF mass spectrometer (Thermo, USA) equipped with a Nanospray Flex source (Thermo, USA). Samples were loaded and separated by a C18 column (15 cm × 75 µm) on an EASY-nLCTM 1200 system (Thermo, USA). The flow rate was 300nL/min and linear gradient was 75 min (0~63 min，5-45% B；63~65 min，45-90% B；65~75 min，90%B; mobile phase A = 0.1% FA in water and B = 0.1% FA in ACN). Full MS scans were acquired in the mass range of 350 – 1500 m/z with a mass resolution of 60000 and the AGC target value was set at 3e6.

The 20 most intense peaks in MS were fragmented with higher-energy collisional dissociation (HCD) with collision energy of 30. MS/MS spectra

were obtained with a resolution of 15000 with an AGC target of 2e5 and a max injection time of 40 ms. The Q Exactive HF dynamic exclusion was set for 30.0 s and run under positive mode.

TMT 10

All analyses were performed by a Q-Exactive HF mass spectrometer (Thermo, USA) equipped with a Nanospray Flex source (Thermo, USA).Samples were loaded and separated by a C18 column (15 cm × 75 µm) on an EASY-nLCTM 1200 system (Thermo, USA). The flow rate was 300nL/min and linear gradient was 75 min (0~63 min，5-45% B；63~65 min，45-90% B；65~75 min，90%B; mobile phase A = 0.1% FA in water and B = 0.1% FA in ACN). Full MS scans were acquired in the mass range of 350 – 1500 m/z with a mass resolution of 60000 and the AGC target value was set at 3e6. The 20 most intense peaks in MS were fragmented with higher-energy collisional dissociation (HCD) with collision energy of 32. MS/MS spectra were obtained with a resolution of 45000 with an AGC target of 2e5 and a max injection time of 80 ms. The Q Exactive HF dynamic exclusion was set for 30.0 s and run under positive mode.

TMT 16 All analyses were performed by a Q-Exactive HF mass spectrometer (Thermo, USA) equipped with a Nanospray Flex source (Thermo, USA). Samples were loaded and separated by a C18 column (15 cm × 75 µm) on an EASY-nLCTM 1200 system (Thermo, USA). The flow rate was 300nL/min and linear gradient was 75 min (0~63 min，5-45% B；63~65 min，45-90% B；65~75 min，90%B; mobile phase A = 0.1% FA in water and B = 0.1% FA in ACN). Full MS scans were acquired in the mass range of 350 – 1500 m/z with a mass resolution of 60000 and the AGC target value was set at 3e6. The 20 most intense peaks in MS were fragmented with higher-energy collisional dissociation (HCD) with collision energy of 32. MS/MS spectra were obtained with a resolution of 45000 with an AGC target of 2e5 and a max injection time of 80 ms. The Q Exactive HF dynamic exclusion was set for 30.0 s and run under positive mode.

iTRAQ

All analyses were performed by a Q-Exactive HF mass spectrometer (Thermo, USA) equipped with a Nanospray Flex source (Thermo, USA). Samples were loaded and

separated by a C18 column (15 cm × 75 μm) on an EASY-nLCTM 1200 system (Thermo, USA). The flow rate was 300 nL/min and linear gradient was 75 min (0~63 min，5-45% B；63~65 min，45-90% B；65~75 min，90%B; mobile phase A = 0.1% FA in water and B = 0.1% FA in ACN). Full MS scans were acquired in the mass range of 350 – 1500 m/z with a mass resolution of 60000 and the AGC target value was set at 3e6.

The10 most intense peaks in MS were fragmented with higher-energy collisional dissociation (HCD) with collision energy of 30. MS/MS spectra were obtained with a resolution of 15000 with an AGC target of 2e5 and a max injection time of 40 ms. The Q Exactive HF dynamic exclusion was set for 30.0 s and run under positive mode.

Database search

Proteome Discoverer (v.2.4) was used to search all of the raw data thoroughly against the sample protein database. Database search was performed with Trypsin digestion specificity. Alkylation on cysteine was considered as fixed modifications in the database searching. For protein quantification method, labeling method was selected. A global false discovery rate (FDR) was set to 0.01 and protein groups considered for quantification required at least 2 peptides.3.6 Fusion Chromatographic conditions versus mass spectrometry conditions (labeling)RPLC analysisRP separation was performed on an 1100 HPLC System (Agilent) using an Agilent Zorbax Extend RP column (5 μm, 150 mm × 2.1 mm). Mobile phases A (2% acetonitrile in HPLC water) and B (98% acetonitrile in HPLC water) were used for RP gradient. The solvent gradient was set as follows: 0~8 min, 8% A; 8~8.01 min, 98%~95% A; 8.01~48 min, 95%~75% A; 48~60 min, 75~60% A; 60~60.01 min, 60~10% A; 60.01~70 min, 10% A; 70~70.01 min, 10~98% A; 70.01~75 min, 98% A. Tryptic peptides were separated at an fluent flow rate of 300μL/min and monitored at 210 and 280 nm. Samples were collected for 8-60 minutes, and eluent was collected in centrifugal tube 1-15 every minute in turn. Samples were recycled in this order until the end of gradient. The separated peptides were lyophilized for mass spectrometry. Mass spectrometry analysis

TMT 6

All analyses were performed by a Fusion mass spectrometer (Thermo, USA) equipped with a Easyspray source (Thermo, USA). Samples were loaded by a capillary trap column (100μm×2cm, RP-C18, Thermo Fisher) and then separated by a capillary analytical column (15 cm × 75μm, RP-C18, Thermo Fisher) on an EASY-nLCTM 1200 system (Thermo, USA). The flow rate was 300 nL/min and linear gradient was set as follow: 0~39 min, 5-31% B; 39~49 min, 31-42% B; 49~54 min, 42-100% B; 54~60 min, 100%B. Full MS scans were acquired in the mass range of 350 – 1800 m/z with a mass resolution of 60000 and the AGC target value was set at 4e5.The 10 most intense peaks in MS were fragmented with higher-energy collisional dissociation (HCD) with collision energy of 35. MS/MS spectra were obtained with a resolution of 15000 with an AGC target of 5e4 and a max injection time of 22 ms. The Fusion dynamic exclusion was set for 60.0 s and run under positive mode.

TMT 10

All analyses were performed by a Fusion mass spectrometer (Thermo, USA) equipped with a Easyspray source (Thermo, USA). Samples were loaded by a capillary trap column (100μm×2cm, RP-C18, Thermo Fisher) and then separated by a capillary analytical column (15 cm × 75μm, RP-C18, Thermo Fisher) on an EASY-nLCTM 1200 system (Thermo, USA). The flow rate was 300 nL/min and linear gradient was set as follow: 0~1 min, 2-5% B; 1~48min, 5~27%B; 48~53 min, 27-35% B; 53~55 min, 35-100% B; 55~60 min, 100%B. Full MS scans were acquired in the mass range of 350 – 1500 m/z with a mass resolution of 120000 and the AGC target value was set at 4e5.The 10 most intense peaks in MS were fragmented with higher-energy collisional dissociation (HCD) with collision energy of 38. MS/MS spectra were obtained with a resolution of 50000 with an AGC target of 5e4 and a max injection time of 86 ms. The Fusion dynamic exclusion was set for 45 s and run under positive mode.

iTRAQ

All analyses were performed by a Fusion mass spectrometer (Thermo, USA) equipped with a Easyspray source (Thermo, USA). Samples were loaded by a capillary trap column (100μm×2cm, RP-C18, Thermo Fisher) and then separated by a capillary analytical column (15 cm × 75μm, RP-C18, Thermo Fisher) on an EASY-nLCTM 1200 system (Thermo, USA). The flow rate was 300 nL/min and linear gradient was set as follow: 0~35 min，5-26% B；35~47 min，26-35% B；47~52 min，35-100% B；52~60 min，100%B. Full MS scans were acquired in the mass range of 350 – 1800 m/z with a mass resolution of 60000 and the AGC target value was set at 4e5.The 10 most intense peaks in MS were fragmented with higher-energy collisional dissociation (HCD) with collision energy of 35. MS/MS spectra were obtained with a resolution of 15000 with an AGC target of 5e4 and a max injection time of 22 ms. The Fusion dynamic exclusion was set for 60.0 s and run under positive mode.

Database search

Proteome Discoverer (v.2.4) was used to search all of the Fusion raw data thoroughly against the sample protein database. Database search was performed with Trypsin digestion specificity. Alkylation on cysteine was considered as fixed modifications in the database searching. For protein quantification method, labeling method was selected. A global false discovery rate (FDR) was set to 0.01 and protein groups considered for quantification required at least 2 peptides.

3.7 QE HF (HF-X) Chromatographic conditions versus mass spectrometry conditions (DIA)

RPLC analysis

RP separation was performed on an 1100 HPLC System (Agilent) using an Agilent Zorbax Extend RP column (5 μm, 150 mm × 2.1 mm).Mobile phases A (2% acetonitrile in HPLC

water) and B (90% acetonitrile in HPLC water) were used for RP gradient. The solvent gradient was set as follows: 0~10 min, 98% A; 10~10.01 min, 98%~95% A; 10.01~37 min, 95%~80% A; 37~48 min, 80~60% A; 48~48.01 min, 60~10% A;48.01~58 min, 10% A; 58~58.01 min, 10~98% A; 58.01~63 min, 98% A. Tryptic peptides were separated at an fluent flow rate of 250μL/minand monitored at 210 nm. Samples were collected for 10-50 minutes, and eluent was collected in centrifugal tube 1-10 every minute in turn. Samples were recycled in this order until the end of gradient. The separated peptides were lyophilized for mass spectrometry.

Mass spectrometry analysis

All analyses were performed by a Q-Exactive HF mass spectrometer (Thermo, USA) equipped with a Nanospray Flex source (Thermo, USA). Samples were loaded and separated by a C18 column (50 cm × 75 μm) on an EASY-nLCTM 1200 system (Thermo, USA). The flow rate was 300nL/min and linear gradient was 90 min (0~60min, 8-25%B; 60~79 min, 25-45% B; 79~80 min, 45-100% B; 80~90 min, 100% B; mobile phase A= 0.1% FA in water and B = 0.1% FA in 80%ACN).

DDA

Full MS scans were acquired in the mass range of 350 – 1650 m/z with a mass resolution of 120000 and the AGC target value was set at 3e6. The 20 most intense peaks in MS were fragmented with higher-energy collisional dissociation (HCD) with collision energy of 27. MS/MS spectra were obtained with a resolution of 30000 with an AGC target of 2e5 and a max injection time of 80 ms. The Q Exactive HF dynamic exclusion was set for 40.0 s and run under positive mode.

DIA

Full MS scans were acquired in the mass range of 350 – 1250 m/z with a mass resolution of 120000 and the AGC target value was set at 3e6.The 32 acquisition windows in MS were fragmented with higher-energy collisional dissociation (HCD) with collision energy of 28 and each acquisition window has 26 m/z. MS/MS spectra were obtained with a resolution of 30000 with an AGC target of 1e6 and a max injection time is set to auto and run under positive mode. Database search Spectronaut was used to search all of the raw data thoroughly against the sample protein database.

Database search

Spectronaut was used to search all of the raw data thoroughly against the sample protein database. Database search was performed with Trypsin digestion specificity. Alkylation on cysteine was considered as fixed modifications in the database searching. Protein, peptide andPSM's false discovery rate (FDR) all set to 0.01. For DIA data, the quantification FDR also set to 0.05. Quantity MS-level was set at MS2.

3.8 Orbitrap Fusion Chromatographic conditions versus mass spectrometry (conditions phosphorylation)

RPLC analysis

All analyses were performed by a Orbitrap Fusion mass spectrometer (Thermo, USA) equipped with a Nanospray Flex source (Thermo, USA).Samples were loaded and separated by a C18 column (15 cm × 75 µm) on an EASY-nLCTM 1200 system (Thermo, USA). The flow rate was 300nL/min and linear gradient was 150 min (0~5 min，3%-7% B；5~125 min，7%-25% B ；125~140 min，25%-35% B；140~145 min，35%-95% B；145~150 min，95%B. mobile phase A = 0.1% FA in water and B = 80% ACN/0.1% FA in water).

Mass spectrometry analysis

Full MS scans were acquired in the mass range of 350 – 1550 m/z with a mass resolution of 60,000 and the AGC target value was set at 4e5. The ten most intense peaks in MS were fragmented with higher-energy collisional dissociation (HCD) with NCE of 38. MS/MS spectra were obtained with a resolution of 15000 with an AGC target of 1e5 and a max injection time of 80 ms. The dynamic exclusion was set for 40.0 s and run under positive mode.

Database search

The LC-MS/MS raw data were imported in MaxQuant for analysis. The database was offered by researchers. The false positive rate of peptide segment identification was controlled below 1%.

3.9 Q-Exactive HF Chromatographic conditions versus mass spectrometry (conditions phosphorylation)

RPLC analysis

All analyses were performed by a Q-Exactive HF mass spectrometer (Thermo, USA) equipped with a Nanospray Flex source (Thermo, USA). Samples were loaded and separated by a C18 column (15 cm × 75 µm) on an EASY-nLCTM 1200 system (Thermo, USA). The flow rate was 300 nL/min and linear gradient was 105 min (0~6 min，5%-8% B；6~91 min，8%-40% B；91~95 min，40%-90% B ；95~105 min，90%B;

mobile phase A = 0.1% FA in water and B = 0.1% FA in ACN).

Mass spectrometry analysis

Full MS scans were acquired in the mass range of 300-1650 m/z with a mass resolution of 120000 and the AGC target value was set at 3e6. The 15 most intense peaks in MS were fragmented with higher-energy collisional dissociation (HCD) with collision energy of 30. MS/MS spectra were obtained with a resolution of 30000 with an AGC target of 1e5 and a

max injection time of 80 ms. The Q Exactive HF dynamic exclusion was set for 30.0 s and run under positive mode

Database search

The LC-MS/MS raw data were imported in MaxQuant for analysis. The database was offered by researchers.The false positive rate of peptide segment identification was controlled below 1%
